# Parametric Optimization for Improving the Machining Process of Cu/Mo-SiC_P_ Composites Produced by Powder Metallurgy

**DOI:** 10.3390/ma14081921

**Published:** 2021-04-12

**Authors:** Emine Şap, Üsame Ali Usca, Munish Kumar Gupta, Mustafa Kuntoğlu, Murat Sarıkaya, Danil Yurievich Pimenov, Mozammel Mia

**Affiliations:** 1 Department of Mechatronics, Vocational School of Technical Sciences, Bingöl University, 12000 Bingöl, Turkey; esap@bingol.edu.tr; 2 Department of Mechanical Engineering, Faculty of Engineering and Architecture, Bingöl University, 12000 Bingöl, Turkey; ausca@bingol.edu.tr; 3 Key Laboratory of High Efficiency and Clean Mechanical Manufacture, School of Mechanical Engineering, Shandong University, Ministry of Education, Jinan 250100, China; munishgupta@sdu.edu.cn; 4 Department of Automated Mechanical Engineering, South Ural State University, Lenin Prosp. 76, 454080 Chelyabinsk, Russia; danil_u@rambler.ru; 5 Mechanical Engineering Department, Technology Faculty, Selcuk University, 42130 Konya, Turkey; mkuntoglu@selcuk.edu.tr; 6 Department of Mechanical Engineering, Sinop University, 57000 Sinop, Turkey; msarikaya@sinop.edu.tr; 7 Department of Mechanical Engineering, Imperial College London, South Kensington, London SW7 2AZ, UK

**Keywords:** Cu/Mo-SiC_P_, metal matrix composite, turning, machinability, parameter optimization

## Abstract

The features of composite materials such as production flexibility, lightness, and excellent strength put them in the class of materials that attract attention in various critical areas, i.e., aerospace, defense, automotive, and shipbuilding. However, the machining of composite materials displays challenges due to the difficulty in obtaining structural integrity. In this study, Cu/Mo-SiC_P_ composite materials were produced by powder metallurgy with varied reinforcement ratios and then their machinability was investigated. In machinability experiments, the process parameters were selected as cutting speed (v_C_), feed rate (f), depth of cut (a_P_), and reinforcement ratio (R_R_). Two levels of these parameters were taken as per the Taguchi’s L8 orthogonal array, and response surface methodology (RSM) is employed for parametric optimization. As a result, the outcomes demonstrated that R_R_ = 5%, f = 0.25 mm/rev, a_P_ = 0.25 mm, v_C_ = 200 m/min for surface roughness, R_R_ = 0%, f = 0.25 mm/rev and a_P_ = 0.25 mm and v_C_ = 200 m/min for flank wear and R_R_ = 0%, f = 0.25 mm/rev, a_P_ = 0.25 mm, v_C_ = 150 m/min for cutting temperature for cutting temperature and flank wear should be selected for the desired results. In addition, ANOVA results indicate that reinforcement ratio is the dominant factor on all response parameters. Microscope images showed that the prominent failure modes on the cutting tool are flank wear, built up edge, and crater wear depending on reinforcement ratio.

## 1. Introduction

The increasing demand for new generation materials with a high strength/weight ratio in parallel with technological developments pushes researchers to do more studies [1,2,3,4]. Composite materials that can meet this demand are increasingly employed among a variety of industries in the mechanical engineering field [5,6]. The main purpose in manufacturing composite materials is to improve some characteristic features of the produced part according to service conditions [7,8]. It may be about surface quality, higher strength, and toughness, which provide longer life and less maintenance need [9,10]. Despite their numerous advantages of composing different types of elements, it brings some challenges with incorporating remarkably hard particles [11,12]. From this side, the machining of composite materials differs from the conventional material machining due to their uncertainties in structural integrity, anisotropy, and abrasiveness [13,14].

Copper is an element covering excellent heat and electrical conductivity and good corrosion resistance prioritized by automotive, informatics, and aerospace industries in order to fabricate lightweight parts [15,16]. The mixture of this alloy with hard and durable particles can improve the wear resistance and mechanical properties [17]. Molybdenum (Mo) and Silicium (Si) carbide particles, which are frequently preferred reinforcement materials among metal matrix composite materials, strengthen the matrix structure and offer high strength and toughness, and good wear resistance as well as thermal properties [18,19]. However, the type and proportion of additive particles have a serious impact on the machinability performance of the material. Various types of main and additive elements have been previously considered for developing specialized composite materials. Some studies showing their effect on turning operations are summarized in Table 1. When the investigated response parameters are taken into consideration, tool wear, surface roughness, and temperature have been highly chosen among machining variables. Depending on varying reinforcement ratios and materials, additive particles bring significant benefits with respect to machinability characteristics. This table outlines the general view of each study including the focal points regarding with this paper. Additionally, according to Razavykia et al. [20], including additive element into the main matrix leads to an increase in interfacial bond between particles and reduces built up edge tendency. Therefore, less change in tool geometry produces smooth surfaces. Barzani et al. [21] showed that material structure changes from coarse to fine with additive and decreased tool wear that is obtained with minimized coefficient of friction between tool and material. Laghari et al. [22] researched tool wear and surface roughness with RSM for optimization and analysis during turning of Al/SiC_P_ composites. Accordingly, feed rate and cutting speed were found as dominant parameters on tool life and surface roughness, respectively. Liu and Zong [23] reported that flank wear can be predicted with a low error rate during turning of SiC_P_ reinforced 2024Al matrix composites. Lin et al. [24] investigated hard TiB_2_ ceramic particles reinforced 7050 Al metal matrix composites. The findings demonstrated that increasing tool wear increases residual stress on the machined surface and transferred into the subsurface with more tool wear.

Balasubramanian et al. [25] made an endeavor for exploration of the machinability of LM6/SiC_p_ composite materials while measuring cutting temperature, vibration, and surface roughness. Cutting speed was the effective factor on cutting temperature and, in addition, feed rate and cutting speed had impacts on surface roughness. Kumar et al. [26] revealed that increasing the amount of additive decreases built up edge while it increases surface roughness. Barzani et al. [27] reported that Bi content reduces built up edge formation with decreasing chip thickness, and also had positive effect on surface roughness. Kumar and Chauhan [28] found that graphite contribution had a positive impact on the machinability of Al7075 material, which represented better surface roughness. Kumar et al. [29] reported that n-B4C and MoS_2_ particles negatively affect the surface roughness of Al2219 composite material due to the built up edge formation and high cutting forces. Pugazhenthi et al. [30] reported that increasing TiB_2_ content deprives surface quality due to the pulling out of this hard particles. Additionally, increasing hard particle content enhances nose wear and diminishes built up edge formation.

In addition to outlined studies, different approaches have been tried for particle reinforced composite materials which need to be analyzing here considering key findings for clear understanding of their turning mechanisms. For example, Bai et al. [31] investigated SiC_P_/Al composites using different cutting tools and cooling conditions while turning in order to research tool wear and surface roughness. Shoba et al. [32] showed that increasing SiC reinforcement gradually decreases the cutting force components due to the dislocation density between reinforcement and matrix materials. The generation of built up edge wear was found to be the primary reason for high cutting forces. Laghari et al. [33] carried out optimization and analysis of SiC_P_ reinforced by 45% of Al composites during turning and utilized RSM for cutting force components. According to their findings, turning behavior of this material was similar to machining of metals. They were also stated that increasing content of SiC particles increased cutting forces. Nikham et al. [34] used different cutting tools for machining Ti metal matrix composites in order to analyze flank wear, cutting forces, and surface roughness. They indicated that high flank wear obtained under high cutting speed values as a result of elevated temperatures. This situation also produced high cutting forces and poor surface roughness. Josyula and Narala [35] measured the performances of cooling conditions on turning of Al-TiCP composites by considering tool wear mechanisms, flank wear, and cutting forces. Nataraj and Balasubramainan [36] performed parametric optimization during turning for ash and SiC_P_ reinforced Al. Surface roughness showed decreasing behavior with increasing depth of cut according to the authors.

Since the studied composite material Cu/Mo-SiC_P_ has never been produced and machined before, a direct comparison is not possible. In a nutshell, reinforcement has been made a positive effect on machinability, which encourages the academicians and researchers to investigate new materials never experienced before. Herein, a new type of material is exposed to turning operation under different cutting conditions in order to examine the change in surface roughness, tool wear, and cutting temperature. Moreover, optimization of parameters is carried out using RSM and Taguchi method while ANOVA is applied in order to observe the effectiveness of cutting parameters and material structure on responses.

## 2. Materials and Methods

A composite material used in the experiments has been developed in the production process with powder metallurgy technique. The details are included in the previous published work [37]. Before the experimental tests, Taguchi orthogonal array was applied. After that, optimization and analysis approaches were utilized for parametric optimization for cutting temperature, flank wear, and surface roughness. This section summarizes whole experimental procedure, utilized tools and materials, approaches, and methods. It is important to demonstrate the details of materials and methods for the future studies to make clear comparison.

### 2.1. Composite Materials Production Process

In the present study, copper is selected as the main matrix material and reinforced with Mo and SiC_P_ particles with the average sizes of 44 µm and between 45–75 µm, respectively. Composites with the dimensions of Ø20 mm × 55 mm were produced by powder metallurgy method. Test samples were prepared with different reinforcement ratios of Mo and SiC_P_ such as 0 wt%, 5 wt%, 10 wt%, and 15 wt%. Scanning electron microscope (SEM) images of powders used in composite production can be seen in [37]. Three of the metallic particles were first mixed for approximately 180 min, then exposed to cold press for about 5 min and finally sintered for 60 min at 1050 °C during the production process. Inert gas (Argon) was used by sintering operation. The amount of copper context gradually decreases with more additives and the ratios of Mo and SiC_P_ increases in percentage. Accordingly, with decreasing of copper ratio, accompanied hard particles enhance total hardness of the part. Herein, hardness measurements were performed as macro hardness on tester device (model: BMS) during 10 s less than 10 kg load. These measurements were repeated 5 times to check the validity of the responses on the surface. The results of SEM and EDS (Energy-dispersive X-ray spectroscopy) analyses for composite materials produced at the end of this process can be seen in [37].

### 2.2. Experimental Procedure

This work is an extension of the authors’ previous published paper [37]. The experiments were performed on a CNC lathe (Goodway GLS-200, Taiwan) under dry machining environment (without any cooling/lubricating means). Before the tests, 0.5 mm layer was removed from the surface for preserving the stability of the cutting operation. Longitudinal turning was carried out to machine the cylindrical shaped samples, and the diameter was reduced from 20 mm to 12 mm during an experiment. Each experiment was repeated three times to obtain the reliable results. Carbide cutting tools defined by CNMG 120,408 GT were utilized in turning tests. The code of the tool holder on which the cutting tools are mounted is DCLNR 2525M12. In order to specify the cutting parameters, preliminary tests were carried out in the direction of the suggestions of the manufacturer of carbide cutting tools (Korloy, South Korea). Therefore, two levels of cutting speeds (150 and 200 m/min), feed rates (0.25 and 0.3 mm/rev), and depth of cut (0.25 and 0.5 mm) were selected as the cutting parameters. During preliminary tests, it was avoided from challenging conditions such as uncontrollable chips and chatter vibrations which can deteriorate the performance of machining. In each experiment, a new cutting tool insert was used for machining of a new sample.

**Figure 1:**
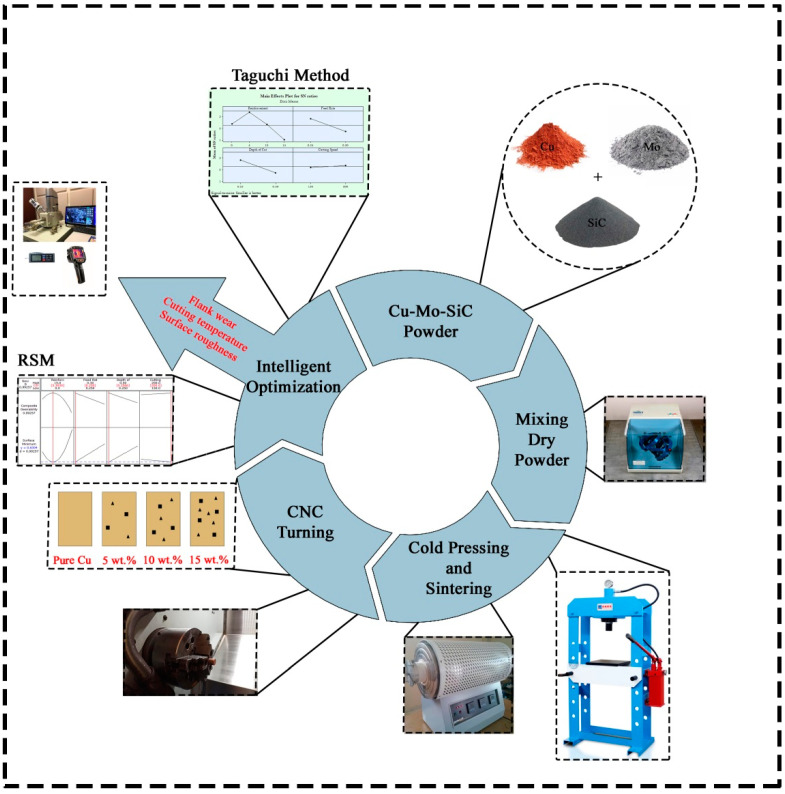
Experimental setup.

In order to measure the machinability characteristic of produced composite samples, three machining responses, i.e., flank wear, surface roughness, and cutting temperature were integrated into the experimental design. Flank wear measurements were performed on microscope device (produced by Leica, Wetzlar, Germany). A thermal camera (Testo 871, Beijing, China) was utilized for cutting temperature measurements. Surface roughness measurements were executed with tracer-tipped tester (TIME3200, Beijing, China). Roughness profile was identified with average surface roughness value, Ra which is highly referred in the past [38,39] because of reliability and representing ability of the quality of scanned length. The measurements were repeated 5 times for each machined sample and the mean of 3 measurements was considered by ignoring the lowest and highest measurement results to avoid possible high deviations. Before the tests, the surface roughness instrument was calibrated with calibration blocks to prevent possible deviations. The experimental setup is provided in Figure 1.

### 2.3. Taguchi Based Design of Experiment and Parameter Optimization

Taguchi is an adopted design method that is widely used in manufacturing applications to reduce time and cost. Taguchi application proposes orthogonal arrays for minimized experiment number according to parameters and their levels. The superior feature of Taguchi method is qualified design ensured by itself [40,41]. Another reason for preference of the method is reliable planning strategy bringing improved efficiency. There are two main tools used by Taguchi to reduce the effect of noise factors and to measure the deviation of optimal value. Signal to noise (S/N) ratio used by Taguchi allows for selecting the appropriate equation for obtaining a response parameter in desired range. This range can be easily defined from the results and using an objective function, optimization is applied for minimization and maximization. Selection of the objective function equation depends on the desired outputs from evaluated response parameter. Since the minimum value of cutting temperature, flank wear, and surface roughness is targeted in metal removal processes, the smallest is best equation (Equation (1)) was used as follows:

(1) $${S/N}_{\mathrm{smaller}\;\mathrm{is}\;\mathrm{the}\;\mathrm{better}}\;=\;-10\log\left[\frac{1}{n}\;\sum_{i=1}^{n}y_{i}^{2}\;\right]$$

In this paper, instead of 32 experiments, with the help of Taguchi L8 orthogonal array, numbers of experiments are reduced. Total 8 experiments were performed including reinforcement ratio, cutting speed, feed rate, and depth of cut with two levels as the input parameters. Table 2 represents Taguchi based experimental design according to L8 orthogonal array.

### 2.4. Parameter Optimization with Response Surface Methodology (RSM)

RSM is an embedded algorithm covers modeling, analysis, and optimization techniques altogether. RSM, which was also used in present study, became popular in the last years as it enables making modeling and optimization at once [42,43]. This method allows for defining the inputs and outputs as independents and dependents, respectively. Consulting the relationship between inputs and outputs, optimum solutions can be derived for individual or total ones. During the modeling process, each input is defined remarking the minimum values. Outputs are also described, with their ranges showing upper and lower boundaries. Each output should be assigned with weight and importance coefficients which demonstrate the importance of the handled parameter. It is needed to determine the target value in terms of the expectations such as minimization or maximization. Optimization results are listed with a desirability value which represents the appropriate model for each response. The desirability value (D) ranges from 0 to 1, and D = 0 represents the worst acceptable value (undesirable), while D = 1 represents the best value (desired target). It was also noted that the desirability value for optimization should be greater than 0.5 [44].

### 2.5. Analysis of Variance (ANOVA)

ANOVA addresses the amount of the effectiveness of design parameters on response parameters. The prominent advantage of this method is providing the effect of factors in numerical forms using different calculation methods based on statistical analysis. Therefore, it is valuable to observe the effect of each parameter in different approaches. Mainly, percent contribution is found using sum of squares, which is calculated with the difference between mean value and measured value. Percent contribution is a good indicator, however, *p*-value demonstrates the importance of the selected parameter whether or not it is in the confidence interval (95%). F value is also a supporting result for the other calculations in accordance with their dominance on response parameter. The above mentioned statistical parameters are generally accepted and give consistent results in a correctly prepared analysis. On the previous studies in machining, an excessive amount of researchers preferred ANOVA-based analysis [45,46]. In this study, ANOVA was implemented to response parameters in order to determine the effect of input parameters.

## 3. Results and Discussion

**Figure 2:**
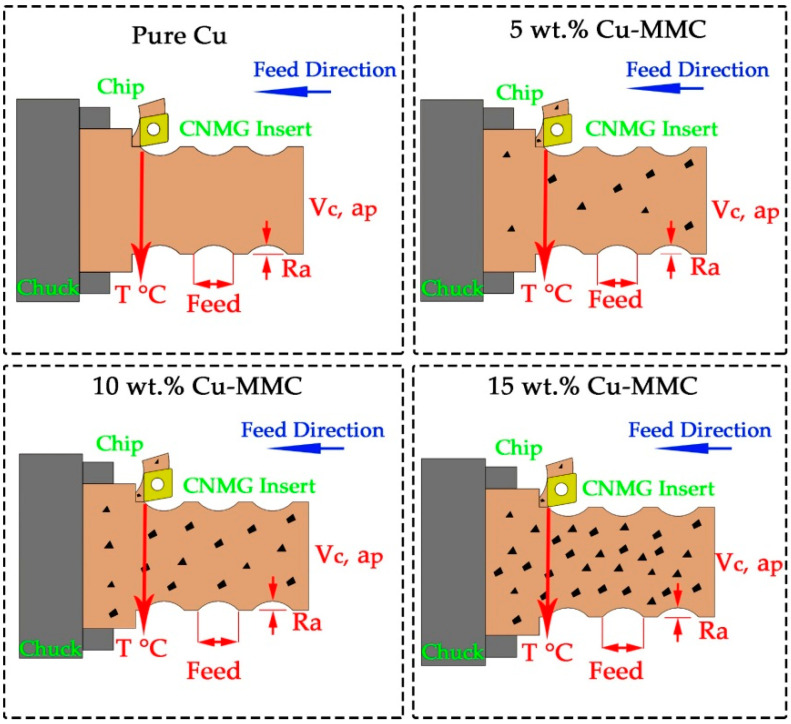
A schematic view of turning of composites.

Unlike conventional metal or alloy turning, some restrictions in terms of unexpected wear patterns make composite turning difficult due to the hard particles randomly dispersed in the body [47]. Actually, interrelations between quality parameters such as surface roughness, vibrations, cutting forces and power, temperatures, and tool wear make this situation more complicated [48]. Namely, concentrated and uniform additive or matrix material in a distinct zone in the body may lead to unusual chip formation for a length of time. This procedure generates suddenly extraordinary cutting conditions which change stress distribution in the contact zones at tool-chip-workpiece. These interfaces are responsible for heat transfer in order to prevent excessive temperature increase on cutting tool and workpiece. Otherwise, structural integrity may be deteriorated, which causes premature adhesive, abrasive, or diffusion wear mechanisms on cutting tool surfaces [49]. Therefore, predetermined cutting tool and workpiece contact conditions begin to change, and the tool geometry is corrupted. After this period, thermal and fatigue loads distinguish which further affect the cutting forces. Meanwhile, due to the unstable cutting during turning, a rise in chatter vibrations can occur [50]. Spoiling effect of cutting forces and vibrations leads to changes in surface integrity of the machined workpiece. The disadvantages mentioned are occasional for each material, but they are more important for composite materials due to the long experimental production process. This pushes the researchers towards taking extra precautions, such as monitoring the cutting process with several sensors and the measuring machinability variables in multiple ways. A clearer understanding of the underlying mechanism can be achieved by comparing different levels of variables. A schematic view of turning of composites having different reinforcement ratios is depicted in Figure 2.

As outlined before, Taguchi based experimental design was implemented, and a total of 8 physical tests were performed including cutting speed, feed rate, and depth of cut in addition to reinforcement ratio. Tool wear, cutting temperature, and average surface roughness were handled as response parameters. Obtained results for each experiment are listed in Table 3. Detailed results of each response output and discussion are presented in the subsections of this section.

### 3.1. Surface Roughness Analysis and Parameter Optimization

Due to its ability to reflect machining success, surface roughness is a good indicator for evaluating the final part quality after turning. This is a universally accepted quality parameter because the surface texture is a prerequisite for delivering a machine element that meets the accepted standards. Among other types, Ra is the most popular one as it calculates the average value of roughness in the measured length [51]. Surface roughness is a sensitive variable related with the harsh conditions as a result of high temperature and pressure at the cutting area. It means the stability of the cutting tool during the movement of surface should be constant as far as possible. Therefore, changing cutting forces, increased vibration, and excessive tool wear have negative effects on surface roughness. Different from the conventional metal/alloy turning, in the present study, the included hard particles make the material structure non-homogeneous throughout the body. This may cause accumulation of hard carbides, which ruptures the tool material and deteriorate the tool and workpiece surface.

**Figure 3:**
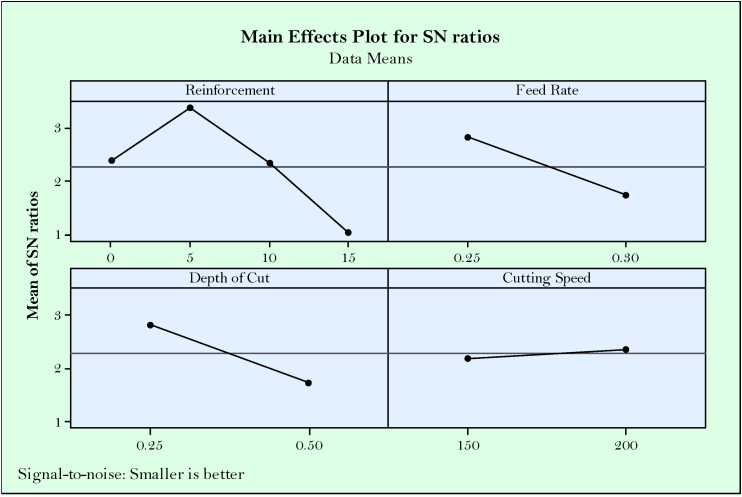
Main effect plots for S/N ratios of surface roughness.

Accordingly, from the main effect plots of S/N ratios shown in Figure 3, it can be concluded that the effect of reinforcement ratio is higher than the effect of cutting parameters. In other words, reinforcement ratio looks like the most effective parameter on surface roughness followed by feed rate, depth of cut, and cutting speed, respectively. Herein, the excessive reinforcement ratio (>5%) reduced the surface roughness due to the difficulty of cutting hard particles and negative effects on the smoothness of the surface. On the other hand, no reinforcement produces a surface worse than 5% reinforcement, and this is mostly due to the joining of the particles as a result of the temperatures at cutting area and ductility behavior of the material. It is understandable that lower feed rate is desirable in accordance with the known equation between surface roughness and the square of feed rate (Ra = f^2^/32*r) [52]. On the other side, increasing cutting speed makes it easier to cut the high hardness particles, which result in better surface quality [20]. This can be attributed to the fact that rising temperatures in parallel with the increased cutting speed thermally soften the workpiece. According to the Taguchi’s main effect plot for S/N ratios, the optimized parameters for surface roughness are R_R_ = 5%, f = 0.25 mm/rev, a_P_ = 0.25 mm, and v_C_ = 200 m/min.

**Figure 4:**
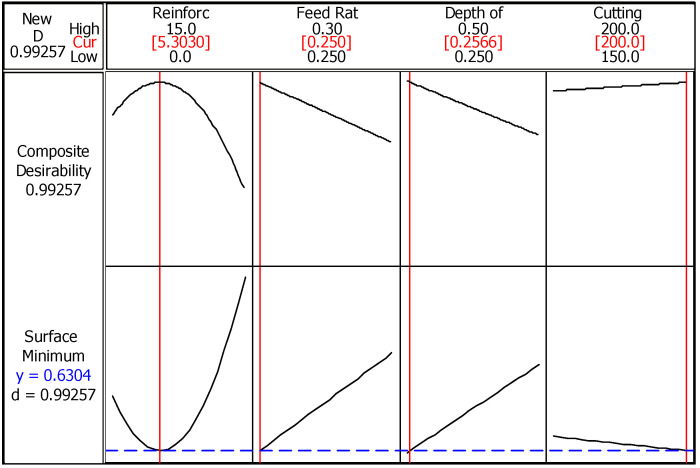
RSM (response surface methodology) optimization of surface roughness.

The second optimization algorithm, RSM, allows the determination of the optimum values of the design parameters for the best response option. Therefore, it is intended to settle the favorable values in the range assigned in modeling stage. Figure 4 summarizes the optimum parameter design obtained from RSM for the best surface roughness value. Here, red lines in each section of input parameter represent optimum value in the range, while black curves indicate trends of both composite desirability and minimum surface roughness. The blue line in this case demonstrates minimum surface roughness value (Ra = 0.6304 µm) at determined conditions. The offered results are close to Taguchi method and the composite desirability shows that the achieved value is highly desirable (D = 0.99257). In brief, same trends are obtained with Taguchi method which reveals the robustness of production and turning processes. Equation (2) presents the calculation procedure of surface roughness via response surface methodology.

*Ra* = −0.561 − 0.0.129 · *R_R_* + 4.65 · *f* + 3.008 · *a_P_* − 0.00031 · *vc* + 0.00208 · *R_R_*^2^ − 0.0368 · *R_R_* · *a_P_* − 9.68 · *f* · *R_R_* (2)

According to the ANOVA results listed in Table 4, reinforcement ratio (67.36%) has dominance impact on surface roughness followed by feed rate (29.23%). The effect of depth of cut and cutting speed can be neglected. Meanwhile, according to the F values, feed rate has more influence on surface roughness because of the degree of freedom which depends on the factor level. In other words, despite having less level compared to reinforcement ratio, feed rate has impressive influence, which can be observed when considering the F value. This also indicates the importance of collaboration in terms of handling the different approaches such as optimization and analysis at the same time. In the point of view of metallurgy, with increasing reinforcement ratios, due to hard-to-cut particles, scraping takes place in some area of surface. Therefore, especially for the Mo-SiC_P_ particles were broken and ruptured from material surface, which produce rougher morphology eventually. Additionally, owing to the feed rate is a movement parameter in the direction of surface roughness production, its variation fluctuates stress distribution on the contact area between tool and workpiece. As a result of random fabrication of particles in the part structure, increasing reinforcement ratio, and feed rate cause lower surface quality.

### 3.2. Tool Wear Analysis and Parameter Optimization

**Figure 5:**
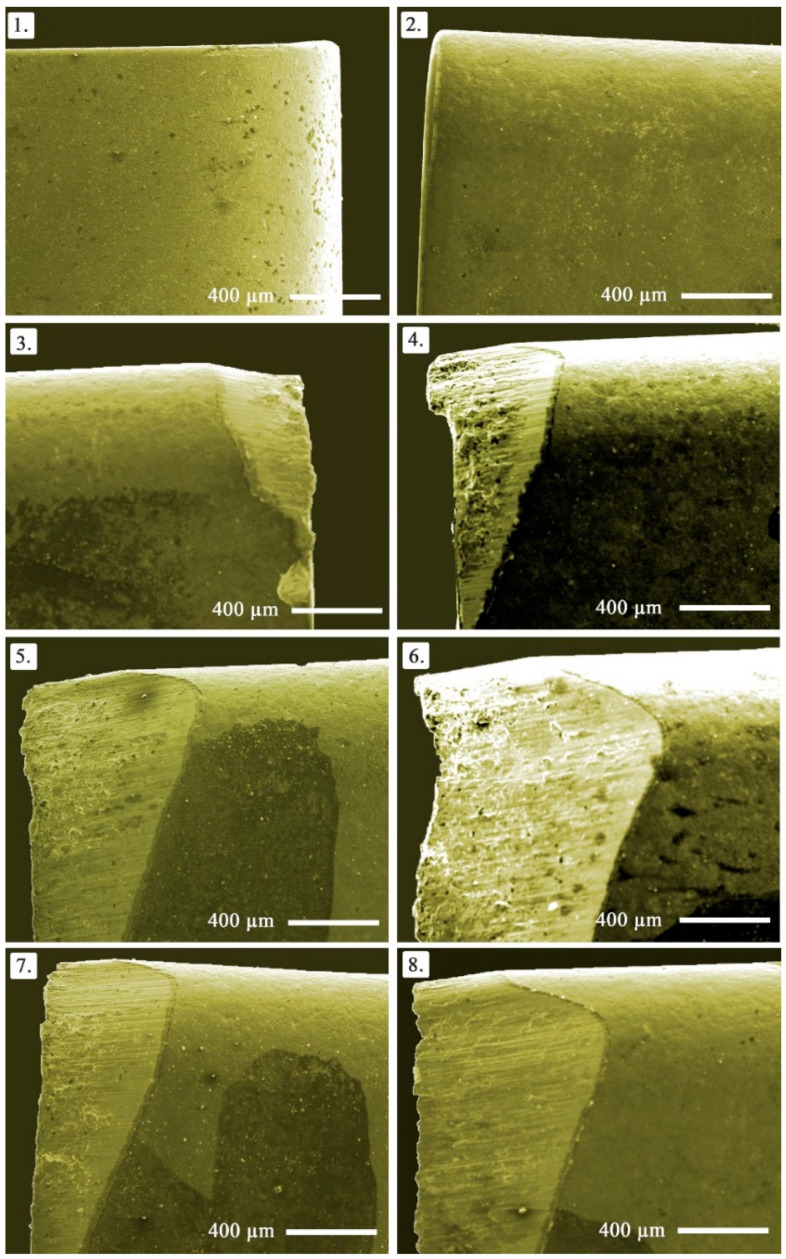
Cutting tool images showing gradual progress in flank wear (Note that the numbers show the order of the test based on design of the experiment).

Among tool wear types, flank wear is the primary one providing wear development information from the main cutting edge and clearance face of the tool. It is also generally accepted and standardized wear indicator in order to determine the remaining useful life of cutting tool [53]. Thus, flank wear becomes the prominent factor in machinability investigations. In this paper, progress in flank wear on the cutting tools is depicted in Figure 5. The main principle in determination of the amount of flank wear depends on the spreading form on the clearance face. Namely, the size of the worn zone can vary along the main cutting edge depending on cutting loads, temperature, tool material, and strength [49]. In the process of determining the amount of wear, there are two options that are frequently considered, namely the maximum depth of wear (VB_max_) and the average wear value (VB). In the present work, maximum wear value was embraced as the amount of wear.

**Figure 6:**
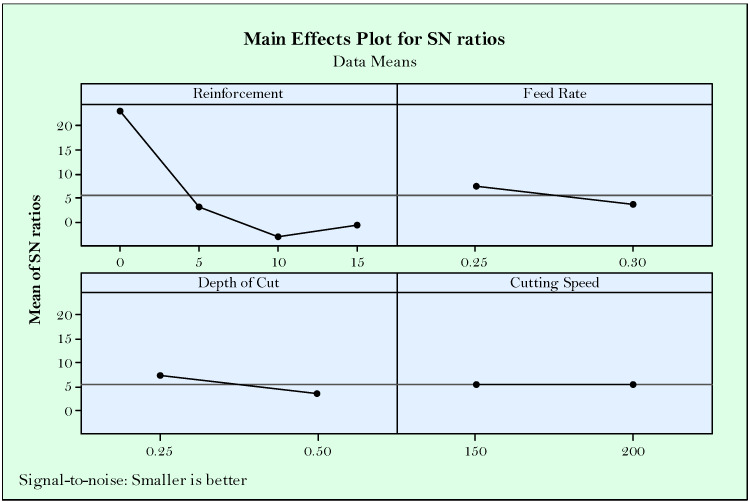
Main effect plots for S/N ratios of flank wear.

Essentially, flank wear develops with higher cutting speeds as it was experimentally tested before in a plenty of studies [43,50,54] in the conventional material turning. However, it should be considered as well that flank wear is triggered by the abrasive wear mechanism ruptured from the cutting tool and workpiece. In the presented paper, the latter comes forward when evaluating the effects of turning parameters in addition to reinforcement ratio. In this context, it is expected the increased hardness in workpiece material makes chip removing more difficult due the difficulty of plastic deformation. Besides, abrasive Mo and SiC_P_ particles teared in the form of chips and fragments sweeping through the tool-workpiece contact areas triggers flank wear. Therefore, in the handled cutting mechanism, reinforcement ratio seems highly effective on flank wear as it is shown in Figure 6. Seemingly, more reinforcement has negative influence, however better flank wear can be obtained at the minimum (5%) ratio. Cutting speed has no observable effect on flank wear, which can be attributed to the implemented range. In other words, selected cutting speed range slightly changes the flank wear. Additionally, lower feed rate and depth of cut are desirable, but less effective compared to reinforcement ratio. According to the main effects plots, optimum solutions for minimum flank wear are R_R_ = 0%, f = 0.25 mm/rev, a_P_ = 0.25 mm, v_C_ = 150 or 200 m/min.

**Figure 7:**
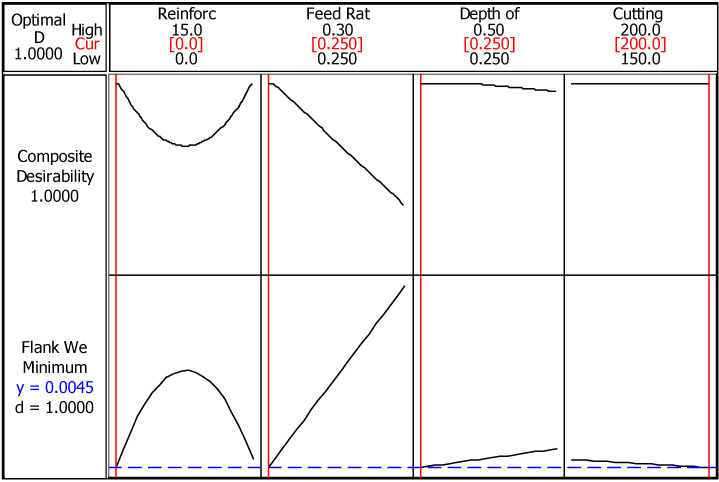
RSM optimization of flank wear.

RSM based optimization for flank wear is demonstrated in Figure 7. The effectiveness of the feed rate is more observable according to this solution. Since feed rate is a kind of speed parameter, high values increase the coefficient of friction and also cutting temperature between the tool and workpiece which is being a contributor to accelerated wear [34]. Additionally, high depth of cut and feed rate compose high material removal rate, which result in forcible cutting, then high cutting forces produce more tool wear. The coincidence of the optimization line and parameter value curve points out the same group of parameters with Taguchi method. According to the best flank wear value (0.0045 mm), the selected parameters are suitable when considering both experimental plan (0.053 mm) and composite desirability (1.0). When it is compared with the surface roughness results, findings imply the same cutting parameters, i.e., feed rate, depth of cut, and cutting speed. In the perspective of reinforcement ratio, it is recommended that since the part quality is accepted as a prominent indicator, the regulations should be arranged according to the surface roughness. Equation (3) presents the calculation procedure of flank wear via response surface methodology.

The effectiveness of the input parameters is listed in the ANOVA results as provided in Table 5. According to the evaluation, reinforcement (96.39%) is the most commanding factor on flank wear considering the percent contribution. Although other parameters such as feed rate with 3.22%, depth of cut with 0.36, and cutting speed with 0.03% have no serious effect on tool flank wear in accordance with percent contribution value, *p* value indicates that all parameters have statistical influence. This can be explained with mentioned optimization results, which can be attributed to their short ranges. In the context of metallurgy, as with the increment of hard particle reinforcement, some unconsolidated particles in the body lead to pores in the structure and cause instant load and discharge of the cutting tool during cutting. This produces fluctuations in the cutting forces and expedites cutting tool wear. In a word, there are two levels in a short range for these parameters, and they are effective in the selected range, which can be determined with *p* value.

*VB_max_* = 1.17194 + 0.22125 · *R_R_* + 0.27925 · *f* − 0.03725 · *a_P_* − 0.02425 · *vc* − 0.58444 · *R_R_*^2^ + 0.19575 · *R_R_* · *a_P_* − 0.29125 · *f* · *R_R_* (3)

**Figure 8:**
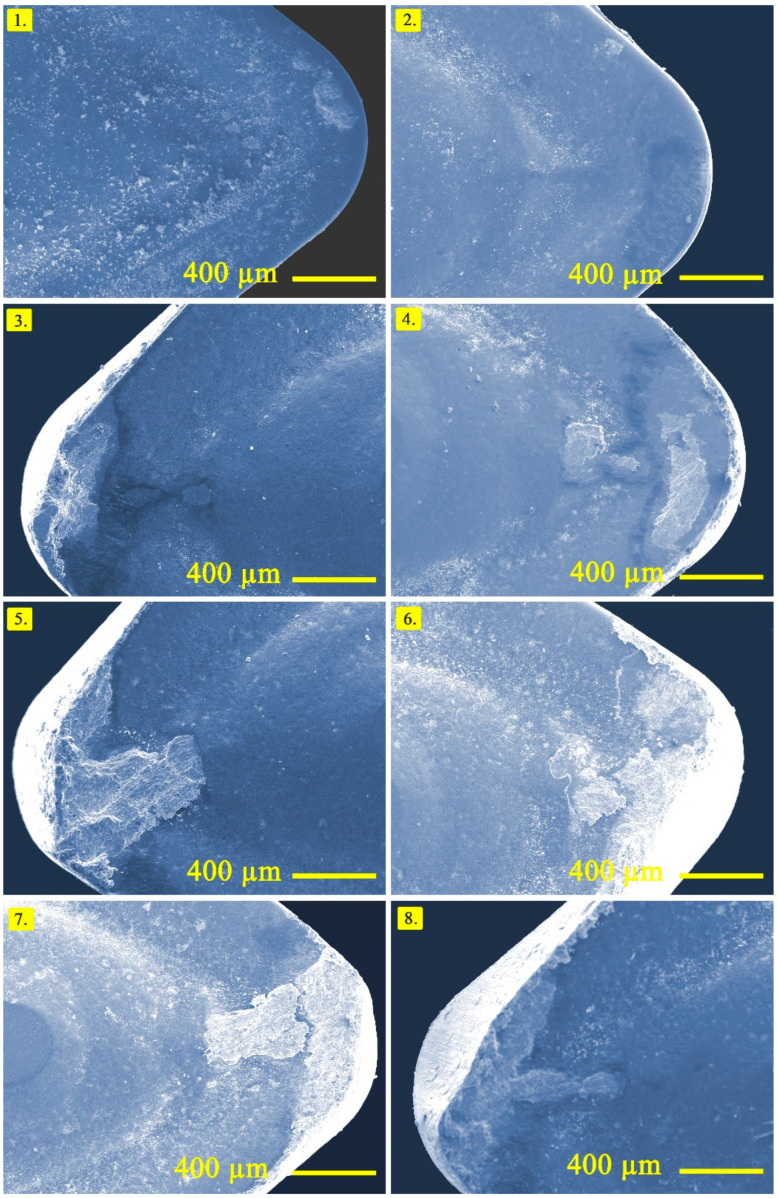
Optical images obtained from the rake face of cutting tools (Note that the numbers show the order of the test based on design of experiment).

When the rake face is considered during turning of composites, built up edge and crater wear seem to be the failure modes during turning of Cu/Mo-SiC_P_ composite materials as shown in Figure 8. In the number of the experiments of 2–6 built up edge formation can be observed, which is connected to the reinforcement ratio. After a certain value of reinforcement, no built up edge formation is observed, however, tool breakage can be seen in the experiment number of 8. This is due to the hard particles and heavy cutting conditions as a result of high level of parameters. In the experiments containing built up edge formation, adhesion wear mechanism is dominant as expected from the ductile material structure. Additionally, crater wear development is clearly seen at numbers 4–8 of the experiments. This is the outcome of the result of high cutting temperature and abrasive wear mechanism triggered mainly by the reinforcement ratio. In addition, a high amount of hard particles in the materials contributed to the excessive crater wear as well as flank wear. Moreover, chipping can be observed in some of the tools, but cannot be attributed directly to the cutting conditions. It is useful to explain in here that non-homogeneous material structure in the composites paves the way for the chipping under some non-traceable conditions.

### 3.3. Cutting Temperature Analysis and Parameter Optimization

**Figure 9:**
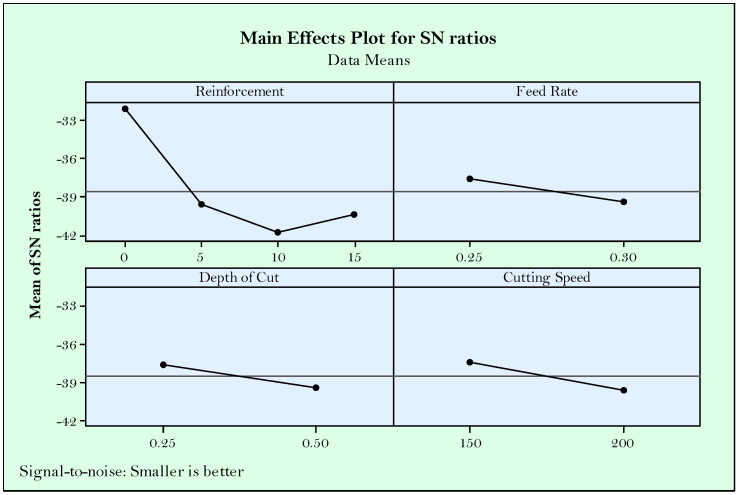
Main effect plots for S/N ratios of temperature.

High pressure and speeds at the cutting area during turning produce heat which further transforms to cutting temperature [55]. The heat generated dissipates among the cutting tool, workpiece, and chip. Most of the heat is removed by chips during a correctly operated cutting process. However, due to the changed cutting geometry and contact conditions, heat accumulation on the cutting tool or workpiece deteriorates material structure with producing residual stresses [56]. Cutting temperature is important for the mentioned reasons and need to be evaluated as a criteria for good machining. Additionally, in the context of this paper, handled responses of surface roughness and flank wear tend to be affected from cutting temperature. It is expected that increasing cutting speed and feed rate increases cutting temperature due to the high friction between materials [57]. Besides, increased material removal by the effect of feed rate and depth of cut lead to stringer force for plastic deformation, ultimately resulting in excessive cutting force and tool wear [45]. In Figure 9, S/N ratios of Taguchi indicate the apparent effectiveness of reinforcement ratio primarily. Similar with the flank wear, hard particles in the materials produced aggravates the cutting event, which produces more heat and directly increases temperature [58]. Herein, reinforcement suppresses the effect of other parameters. Seemingly, no reinforcement is preferable, however, in case of the usage of reinforcement, it seems advantageous in terms of minimized temperature. As seen in Figure 9, except for the reinforcement ratio, cutting parameters approximately have the same influence on temperature. Finally, the same results can be suggested according to Taguchi S/N results for optimizing the flank wear R_R_ = 0%, f = 0.25 mm/rev, a_P_ = 0.25 mm, v_C_ = 150 m/min.

**Figure 10:**
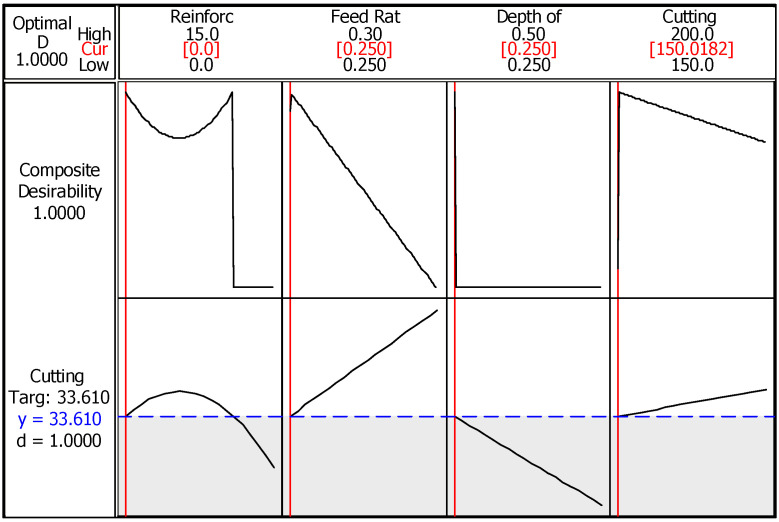
RSM optimization of cutting temperature.

When the results for the minimized cutting temperature are applied to the RSM method, similar solutions with Taguchi are obtained. Figure 10 summarizes the tendencies for production and cutting parameters in order to obtain minimum cutting temperature. First, composite desirability (1.0) is quite convincing in terms of the temperature value (33.61 °C) which is pending with the experimental result. The effects of the other parameters are obvious and can be neglected, except for the reinforcement ratio. In Table 6, ANOVA for S/N ratios of cutting temperature is listed. Briefly, percent contribution ratio of reinforcement (79.69%) has a higher effect in comparison with the other parameters. According to *p* and F values, cutting speed, feed rate, and depth of cut still have no influence on cutting temperature. Since the basic mechanisms for explanation of the effect of parameters are mentioned in detail before, there is no need to give them again here. In metallurgical perspective, due to copper acting as filler material in the structure and hard particles consolidates between the matrixes, increasing reinforcement ratio makes the body with high strength. Strong bonding between main and reinforcement materials increases the required cutting force and makes the cutting operation difficult. Therefore, increasing cutting forces led to higher shear stress and turning becomes harder, which elevates the cutting temperatures. It should be noted that measuring of cutting temperature is quite satisfying in terms of using the Cu/Mo-SiC_P_ composite materials in practical applications. Equation (4) presents the calculation procedure of cutting temperature (CT) via response surface methodology.

*CT* = 117.444 + 26.850 · *R_R_* + 42.275 · *f* + 3.050 · *a_P_* + 12.425 · *vc* − 42.919 · *R_R_*^2^ + 50.100 · *R_R_* · *a_P_* − 6.425 · *f* · *R_R_* (4)

### 3.4. Multiple Optimizations of Characteristics

In addition to single optimization of machinability characteristics, multiple optimizations present a global solution for all parameters which can be useful, especially for the manufacturers. For the multiple optimization of cutting temperature, flank wear, and surface roughness, RSM approach was implemented with defining the ranges of each parameter. Then, for the minimization of each, the lowest values were identified and the software was calculated. In Table 7, for each parameter, the ranges are identified, and weight and import values are selected as 1 for the purpose of order of them. In this way, all parameters are set to the same order for guaranteed and standardized effect of inputs. Additionally, it is noteworthy to say that initial conditions are set as R_R_ = 0%, f = 0.25 mm/rev, a_P_ = 0.25 mm, v_C_ = 150 m/min in the modeling process in RSM algorithm.

**Figure 11:**
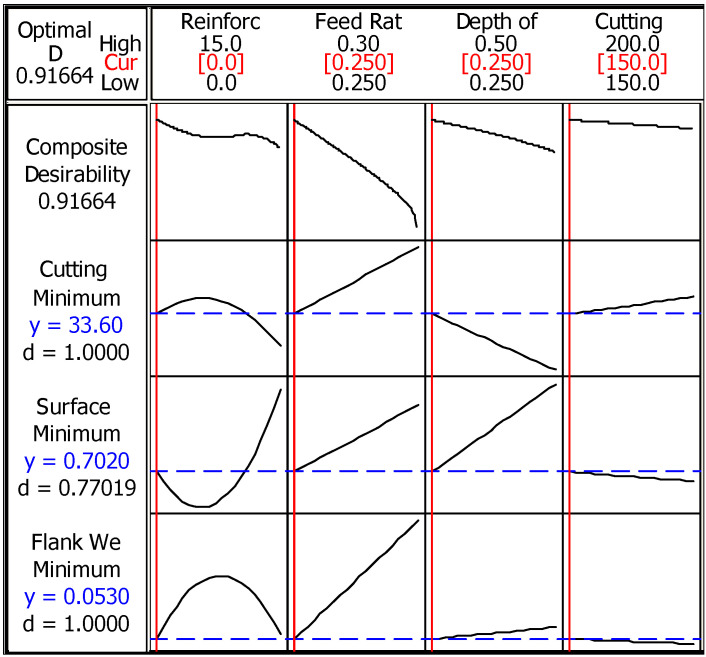
Multiple optimization results for characteristics.

Figure 11 represents the solution and for the optimization/minimization of characteristics, lowest values of reinforcement, feed rate, depth of cut, and cutting speed that need to be selected. Accordingly, when these conditions are applied, temperature and flank wear can be optimized with high accuracy. Total desirability (91.6%) is satisfying, and even the surface roughness seems low (77%). Therefore, multiple optimizations are reliably preferred for minimization of cutting temperature, flank wear, and surface roughness during turning of composites.

A special type of composite materials, Cu/Mo-SiC_P_, was evaluated with statistical analysis and optimization methods such as Taguchi S/N ratios and RSM as multiple and individual optimization. According to the turning experiments, optimal solutions and dominant parameters on machining characteristics were found. A robust design was obtained when considering the harmony of the findings with both optimization technique and ANOVA calculations. In addition, the findings are satisfying in terms of providing the best machining parameters and material combination for the production phase in practice.

## 4. Conclusions

The optimization and analysis of the results demonstrates that a robust design can be developed when it comes to produce and machine Cu/Mo-SiC_P_ composites. It is valuable that the obtained parts can be evaluated in the automotive and aerospace industries when considering the optimum solutions presented here. Surface roughness optimization indicates that second level of reinforcement ratio (R_R_ = 5%), lower levels of feed rate (f = 0.25 mm/rev) and depth of cut (a_P_ = 0.25 mm), and a higher level of cutting speed (v_C_ = 200 m/min) need to be selected according to Taguchi’s S/N ratio results. Since the RSM method also offers a solution in intermediate values, its suggestion is R_R_ = 5.03%, f = 0.25 mm/rev, a_P_ = 0.2566 mm, and v_C_ = 200 m/min. ANOVA analysis results depicts reinforcement ratio (67.36%) is the most effective parameter and feed rate (29.23%) comes the second place. A consensus is obtained from RSM and Taguchi in the parameter optimization for flank wear and cutting temperatures which infers that the optimized parameters are R_R_ = 0%, f = 0.25 mm/rev, a_P_ = 0.25 mm, v_C_ = 200 m/min for flank wear, and R_R_ = 0%, f = 0.25 mm/rev, a_P_ = 0.25 mm, v_C_ = 150 m/min for cutting temperature. The reinforcement ratio with 96.39% and 79.69% contribution rate is the dominant factor for both flank wear and cutting temperature, respectively. It can be stated that the effect of cutting parameters is at a negligible level. It was observed that the wear progress on the flank face occurred regularly due to the abrasive wear mechanism as the reinforcement ratio increased. In addition, it can be said that built up edge and crater wear are the main tool failure types on the rake face of the cutting tool as a result of adhesive and abrasive wear mechanisms. Some chipping and tool breakage developments can be observed in some conditions, which can be attributed to the ratio of hard particles determined by reinforcement ratio. When all responses are taken into consideration, the general tendency is to select the surface roughness based on optimum parameters, which can be compensated from flank wear and cutting temperature. For the future work, different reinforcement ratios in Cu and several types of metals can be tried for manufacturing of composites. Additionally, it is important to indicate the effect of lubrication/cooling conditions on machinability characteristics of this composite, not just for turning, but for milling, drilling, and grinding.

## Figures and Tables

**Table 1:** A literature summary of the turning of the particle reinforced composites.

Ref.	Reinf. Ratios	Main/ Additive Materials	Investigated Response Parameters	The Effect of Increased Reinforcement	The Effect of Other Parameters
[20]	0.4%	Al-Mg_2_Si/Bismuth	Surface Roughness, Cutting Force, Chip Formation, Tool Wear	Lower cutting force, surface roughness, chip length, and built-up-edge tendency	Feed rate and cutting speed are effective on surface roughness
[21]	0.4%	Al-Mg_2_Si/Bismuth	Surface Roughness, Cutting Force, Chip Formation, Tool Wear	Improved cutting force, surface roughness, chip breakability, less built up edge tendency	Feed rate and cutting speed are effective on surface roughness
[26]	5-7-10%	Al-4.5%Cu/TiC	Cutting Force, Surface Roughness, Built Up Edge Formation, Chip Formation	Discontinuous and short chips, less built up edge, poor surface roughness	Increasing feed rate, depth of cut, and decreasing cutting speed have negative impact on surface roughness
[27]	1% Bi- 0.5% Sb	Al–11.3Si–2Cu	Cutting Force, Surface Roughness, Chip Formation	Bi containing has positive, Sb containing has negative effect on surface roughness	Increasing feed rate enhances surface roughness
[28]	10% SiC- 7% SiC and 3% graphite	Al7075	Surface roughness	Graphite particles improve surface roughness	Feed rate provides primary contribution
[29]	2% n-B_4_C- 2% MoS_2_	Al2219	Cutting Force, Surface Roughness	Particle inclusion had negative effect on surface roughness	High cutting speed and low feed rate produce better surface roughness
[30]	0-12% TiB_2_	AA7075	Cutting Force, Surface Roughness, Built Up Edge Formation, Chip Formation	The reinforcement decreases surface roughness	If cutting speed increases, built up edge decreases, surface roughness increases

**Table 2:** Experimental design according to Taguchi’s L_8_ orthogonal.

Experiment Number	Reinforcement Ratio R_R_ (%wt.)	Feed Rate f (mm/rev)	Depth of Cut a_P_ (mm)	Cutting Speed v_C_ (m/min)
1	1	1	1	1
2	1	2	2	2
3	2	1	1	2
4	2	2	2	1
5	3	1	2	1
6	3	2	1	2
7	4	1	2	2
8	4	2	1	1

**Table 3:** Experimental results (Copyrights reserved) [37].

Experiment Number	Reinforcement (%wt.)	Feed Rate (mm/rev)	Depth of Cut (mm)	Cutting Speed (m/min)	Surface Roughness (µm)	Flank Wear (mm)	Cutting Temperature (°C)
1	0	0.25	0.25	150	0.702	0.053	33.6
2	0	0.3	0.5	200	0.824	0.097	48.9
3	5	0.25	0.25	200	0.628	0.541	81.1
4	5	0.3	0.5	150	0.735	0.943	113.5
5	10	0.25	0.5	150	0.744	1.245	93.1
6	10	0.3	0.25	200	0.785	1.699	163
7	15	0.25	0.5	200	0.832	0.955	131.1
8	15	0.3	0.25	150	0.95	1.245	84.5

**Table 4:** Analysis of variance for S/N ratios of surface roughness.

Source	Degree of Freedom	Sum of Squares	Mean Square	F-Value	*p*-Value	Percent Contribution (%)
Reinforcement	3	5.51437	1.83812	29.06	0.135	67.36
Feed te	1	2.39400	2.39400	37.85	0.103	29.23
Depth of cut	1	0.16257	0.16257	2.57	0.355	1.98
Cutting speed	1	0.05459	0.05459	0.86	0.523	0.66
Residual error	1	0.06325	0.06325	-	-	0.77
Total	7	8.18877	-	-	-	100

**Table 5:** Analysis of variance for S/N ratios of flank wear.

Source	Degree of Freedom	Sum of Squares	Mean Square	F-Value	*p*-Value	Percent Contribution (%)
Reinforcement	3	849.475	283.158	3,233,584.61	0.000	96.39
Feed rate	1	28.426	28.426	324,614.34	0.001	3.22
Depth of cut	1	3.216	3.216	36,727.61	0.003	0.36
Cutting speed	1	0.084	0.084	961.68	0.021	0.03
Residual error	1	0.000	0.000	-	-	0
Total	7	881.202	-	-	-	100

**Table 6:** Analysis of variance for S/N ratios of cutting temperature.

Source	Degree of Freedom	Sum of Squares	Mean Square	F-Value	*p*-Value	Percent Contribution (%)
Reinforcement	3	112.572	37.524	4.32	0.337	79.69
Feed rate	1	6.532	6.532	0.75	0.545	4.62
Depth of cut	1	3.288	3.288	0.38	0.649	2.32
Cutting speed	1	10.169	10.169	1.17	0.475	7.21
Residual error	1	8.694	8.694	-	-	6.16
Total	7	141.256	-	-	-	100

**Table 7:** Response surface methodology parameter design.

Parameter	Goal	Lower	Target	Upper	Weight	Import
Cutting Temperature	Minimum	33.6	33.6	131.100	1	1
Surface Roughness	Minimum	0.628	0.628	0.95	1	1
Flank Wear	Minimum	0.053	0.053	1.699	1	1

## Data Availability

Not Applicable.
